# Do Adults with High Functioning Autism or Asperger Syndrome Differ in Empathy and Emotion Recognition?

**DOI:** 10.1007/s10803-016-2698-4

**Published:** 2016-02-16

**Authors:** Charlotte B. Montgomery, Carrie Allison, Meng-Chuan Lai, Sarah Cassidy, Peter E. Langdon, Simon Baron-Cohen

**Affiliations:** Department of Clinical Psychology, Norwich Medical School, University of East Anglia, Norwich, NR7 4TJ UK; Child and Family Psychology Service, Child Development Centre, Hospital Road, Bury St Edmunds, IP33 3ND UK; Autism Research Centre, Department of Psychiatry, University of Cambridge, Douglas House, 18b Trumpington Road, Cambridge, CB2 8AH UK; Department of Psychiatry, National Taiwan University Hospital and College of Medicine, Taipei, Taiwan; Child and Youth Mental Health Collaborative at the Centre for Addiction and Mental Health and the Hospital for Sick Children, and Department of Psychiatry, University of Toronto, Toronto, ON Canada; Centre for Research in Psychology, Behaviour and Achievement, Coventry University, Coventry, UK; The Tizard Centre, University of Kent, Canterbury, CT2 7LR UK; Broadland Clinic, Hertfordshire Partnership University NHS Foundation Trust – Norfolk, Little Plumstead, Norwich, NR13 5EW UK; CLASS Clinic, Cambridgeshire and Peterborough NHS Foundation Trust, Cambridgeshire, UK

**Keywords:** Autism, Asperger, Empathy, Emotion, DSM-5

## Abstract

The present study examined whether adults with high functioning autism (HFA) showed greater difficulties in (1) their self-reported ability to empathise with others and/or (2) their ability to read mental states in others’ eyes than adults with Asperger syndrome (AS). The Empathy Quotient (EQ) and ‘Reading the Mind in the Eyes’ Test (Eyes Test) were compared in 43 adults with AS and 43 adults with HFA. No significant difference was observed on EQ score between groups, while adults with AS performed significantly better on the Eyes Test than those with HFA. This suggests that adults with HFA may need more support, particularly in mentalizing and complex emotion recognition, and raises questions about the existence of subgroups within autism spectrum conditions.

## Introduction

Autism spectrum conditions (ASC) are complex and pervasive neurodevelopmental conditions associated with lifelong difficulties across social, emotional, and behavioural domains (Amaral et al. 2008; Groen et al. 2008; Lai et al. 2013). In recent years, however, the conceptualisation of ASC has changed. The latest edition of the Diagnostic and Statistical Manual of Mental Disorders (DSM-5; APA 2013) has removed the previously discrete diagnostic presentations of Pervasive Developmental Disorders, including Pervasive Developmental Disorder—Not Otherwise Specified (PDD-NOS), Asperger’s disorder and autistic disorder, and subsumed them within one broader, ‘Autism Spectrum Disorder’ (ASD) diagnosis (here, we use Autism Spectrum Conditions (ASC), instead of ASD, and Asperger syndrome (AS) instead of Asperger’s disorder, seeing these terms as synonymous but with ASC and AS as less stigmatising). The World Health Organisation has not yet proposed to do the same in their planned 2017 revision of the International Classification of Diseases (ICD) manual and so the two international diagnostic manuals currently contradict one another. This shift has raised questions as to how ASC are conceptualised; whether a move towards a single broader diagnostic category better reflects natural kinds and, if not, whether this broader categorisation is clinically useful.

Two subgroups that are often grouped together in research designs and clinical service provision are AS and high functioning autism (HFA). AS was a previously discrete diagnosis in the fourth edition of the DSM (DSM-IV; APA 2000), and remains one in the current edition of the ICD (ICD-10; WHO 1994), separated primarily due to the core feature of typical language acquisition. HFA is a term used to describe the clinical presentation of autism without any additional intellectual disability and is not a term used in either the DSM or the ICD. It is, however, a widely used clinical diagnosis to identify individuals on the autistic spectrum who had a history of language delay but do not have the associated difficulties of an intellectual impairment.

The similarities between AS and HFA are well documented. Both conditions present with varying degrees of difficulty in social communication alongside the presence of unusually narrow interests, resistance to change, and highly repetitive behaviours (Volkmar and Partland 2014). Furthermore, group differences between HFA and AS have been argued to be predominantly associated with level of intelligence (Witwer and Lecavalier 2008). Research does, however, suggest that these subgroups may be distinct from one another across other features central to the conceptualisation of ASC (Howlin 2003; Pina-Camacho et al. 2013). There are relatively few studies exploring these differences (Matson and Boisjoli 2008; Planche and Lemonnier 2012) and the results are often contradictory (Lai et al. 2015). In response to changes within the DSM-5, Tsai and Ghaziuddin’s (2014) literature review of comparative studies showed 4 studies concluding that no significant differences exist between HFA and AS, 2 studies concluding that AS was a distinct subgroup of ASC, and 4 studies concluding that there was insufficient support for the removal of AS from DSM-5 at this stage. These conflicting data have limited our ability to draw conclusions as to whether AS and HFA are distinct conditions.

From a cognitive perspective, there is some evidence that people with AS may have superior verbal (VIQ) over performance intelligence (PIQ) while the opposite is the case in those with HFA (Planche and Lemonnier 2012). This is perhaps unsurprising given the atypical language development in HFA, however, the profile is not consistently demonstrated. Multiple studies have found no difference in VIQ between the groups (Spek et al. 2008; Wilson et al. 2014) and other studies show a mixed profile based on individual strengths and difficulties, the pattern of which is not consistent enough to enable diagnostic categorisation (Ghaziuddin and Mountain-Kimchi 2004; Williams et al. 2008). There is, however, growing evidence to suggest that the two conditions can be distinguished at the neuroanatomical level. A meta-analysis of magnetic resonance imaging (MRI) studies of the neuroanatomy of people with AS compared to people with HFA, using voxel-based morphometry, found significant differences in grey matter volume between the groups with distinct distribution patterns (Yu et al. 2011). Although a systematic review of structural MRI data suggested a less clear distinction (Pina-Camacho et al. 2013), the authors concluded that, on the basis of available evidence, it may be too soon to remove different subgroups of ASC from diagnostic manuals as fundamental differences could exist. Indeed, a recent study from our group showed that in male adults with ASC, those with versus without language delay partly differed in terms of brain structure (Lai et al. 2015). This suggests that subsuming these two subgroups into a single over-arching diagnostic category may risk masking the subtle differences in development and outcome (Lai et al. 2013).

There is a lack of research exploring the possible differences in social and emotional processing between people with AS and HFA, despite the importance of this area in informing clinical practice (Palmen et al. 2012). Social difficulties are arguably the most prominent and easily measured ASC trait (Schultz 2005). One hypothesis is that these difficulties are underpinned by a ‘theory of mind’ impairment or a ‘mindblindness’ (Baron-Cohen 1995). Theory of mind refers to the ability to attribute mental states to oneself and others and includes the ability to understand that it is possible for others to hold thoughts and beliefs that are different from your own (Baron-Cohen et al. 1985; Premack and Woodruff 1978). A theory of mind impairment therefore leads to core social difficulties in guessing how others may feel in a given situation and subsequent difficulties in understanding and interpreting social cues.

Research consistently shows that children with ASC develop theory of mind skills later than children who are developing typically and that some people with ASC never acquire a truly implicit theory of mind (Lai et al. 2013; Scheeren et al. 2013). There is also evidence to suggest differences in the development of theory of mind in children with AS compared to children with HFA. Ozonoff et al. (1991) found that children with AS outperformed children with HFA on first-order false belief tasks; suggesting a difference in theory of mind skills between these two subgroups of ASC. Paynter and Peterson (2010) also found theory of mind was significantly more impaired in children with HFA aged 5–12 years of age, compared to children with AS.

Theory of Mind is central to the development of another neurocognitive construct; empathy. The hypothesis of ASC being associated with difficulties in empathising extends the theory of mind hypothesis by considering the impact of this mindblindness on the ability to respond appropriately to emotions in others (Baron-Cohen 2002). Empathy is therefore not viewed as a unitary concept, it comprises both cognitive components and affective components (Sucksmith et al. 2013). Interestingly, there is growing evidence to suggest that people with ASC may show greater difficulties with cognitive empathy (the ability to correctly identify other people’s feelings or beliefs and understand the reasons for these) than affective empathy (the ability to offer an appropriate emotional response to another person’s mental state) (Baron-Cohen 2011; Mazza et al. 2014).

Theory of mind and empathy difficulties underpin the social and communicative difficulties seen in ASC and have an impact on the formation of positive social relationships and interactions (Goldstein and Winner 2012). Atypical social interaction is consistently reported as being one of the earliest observable ASC traits and has been demonstrated in infancy, even before formal diagnosis would be possible (Bedford et al. 2012; McConnell 2002; Rogers 2009). Furthermore, this phenotype is almost exclusively observed in ASC and is not characteristic of other developmental conditions (Schultz 2005). The majority of studies exploring social difficulties to date have focused on child populations, meaning that patterns of social difficulties in later life are not as well understood (Kaland 2011). Even fewer studies have explored social cognition differences between adults with HFA versus AS, but differences in this feature may be an important clinical reason for keeping AS distinct from HFA (Pina-Camacho et al. 2013).

Ghaziuddin (2010) explored social interaction in 39 children with HFA compared to 58 children with AS and reported significantly different social profiles between the groups. Using Wing and Gould’s (1979) social impairment profiles, 79 % of children with AS were rated as being ‘active but odd’ whereas 82 % of children with HFA were identified as falling within the ‘aloof and passive’ category. These findings demonstrate significantly different social profiles in children with HFA compared to children with AS. In adulthood, people with AS have also been shown to have a more ‘active but odd’ social profile than people with HFA who, as with childhood populations, show a more passive social profile (Ghaziuddin 2010).

An active profile will increase social experiences which may in turn increase the opportunity for social difficulties to arise. This difference between adults with AS and HFA may therefore have important clinical implications (Ghaziuddin 2010). Recent studies have shown experiences of suicidal ideation to be more than nine times higher in adults with AS than in the general population in England (Cassidy et al. 2014). This finding in AS populations specifically may be influenced by the degree of insight into social difficulties. Gotham et al. (2014) reported a relationship between a person’s own perception of their autism-related difficulties and depressive symptoms, regardless of the objectively assessed degree of impairment. This suggests that insight into difficulties is a more influential factor in the development of depression than actual social ability. The active but odd social presentation associated with AS may also make this group, in particular, at greater risk of social difficulties and low mood.

Positive social experiences and activities are linked to overall quality of life (Mansell et al. 2002; Schalock 2004; Tobin et al. 2014). The impairments in social interaction and social communication, which are core features of ASC, may thus be causing a lower overall quality of life relative to level of insight (Gotham et al. 2014). Understanding whether differences exist in social-emotional functioning between people with HFA and people with AS may reveal whether social interactions are experienced differently and if we need to tailor support to people differently depending on their ASC subgroup. Further examination of differences between those with HFA and AS may therefore provide a useful test case for the merits of the single ASC diagnosis in DSM-5.

The present study aimed to examine whether (1) adults with HFA versus AS differ in their drive to empathise with others and (2) whether objective differences exist between these subgroups in the ability to ‘read’ mental states in others’ eyes. Sex differences were explored first to consider the impact, or lack of impact, on the main comparisons. The Extreme Male Brain theory of autism (Baron-Cohen 2002) suggests that any sexual dimorphism observed in typical populations in empathy will be attenuated or completely abolished (Baron-Cohen et al. 2014, 2015) in ASC, so no difference in scores between males and females with ASC were predicted in the present study. We predicted that HFA would be associated with greater difficulties in these skills as a result of early developmental language acquisition difficulties and the impact that language has on social skills development (Ozonoff et al. 2000; Howlin 2003).

## Methods

### Participants

43 adults, aged 18 years or older, with HFA and 43 adults with AS were selected for comparison from the Cambridge Autism Research Database (CARD; http://www.autismresearchcentre.com) were included in this study. Participants with AS were selected at random, and stratified by sex in order to ensure that both groups were matched. All participants reported being diagnosed with either AS or HFA by a qualified professional (Clinical Psychologist or Psychiatrist) using DSM-IV (APA 2000) and/or ICD-10 (WHO 1994) criteria at recognised clinics. Self-report of clinical diagnoses has been shown to be very accurate, with agreement as high as 98 %, in the ASC population (Auyeung et al. 2012; Daniels et al. 2012). Participants were matched for intelligence using Raven’s Progressive Matrices (Raven et al. 1997), a non-verbal measure of intelligence, which confirmed the absence of a clinically defined learning disability (IQ < 70) in all participants (Table 1). This is important in investigating AS-HFA differences as previous inconsistent data appears to be highly influenced by variation in IQ (Witwer and Lecavalier 2008).

**Table 1:** Participant demographics

	*N*	*M* age (*SD)*	*M* IQ (*SD*)	*N* male	*N* female
HFA	43	39.09 (13.05)	18.91 (1.74)	20	23
AS	43	37.95 (12.52)	18.91 (1.74)	20	23
Total	86			40	46

### Measures

#### The Empathy Quotient

Participants completed the Empathy Quotient (EQ; Baron-Cohen and Wheelwright 2004), a 60 item self-report questionnaire designed to measure how easily a person can pick up on other people’s feelings and how strongly they are affected by other people’s feelings. The EQ therefore measures both cognitive and affective empathy (Baron-Cohen and Wheelwright 2004). Participants are required to respond to each item by selecting one of four options: ‘strongly agree’, ‘slightly agree’, ‘slightly disagree’ or ‘strongly disagree’. The EQ was developed and validated on adults with both HFA and AS compared to a control group and has been shown through confirmatory factor analysis to have reliability of .93 (Allison et al. 2011). Test–retest reliability of the EQ is also high, at *r* = .835 (*n* = 25, *p* = 0.0001; Lawrence et al. 2004). The EQ is therefore effective in measuring empathy and, as anticipated by the social difficulties associated with ASC, people with ASC consistently score significantly lower on the EQ than people without an ASC do (Baron-Cohen et al. 2014, 2015).

#### The ‘Reading the Mind in the Eyes’ Test

Participants were also compared on the ‘Reading the Mind in the Eyes’ Test: Revised Edition (Eyes Test; Baron-Cohen et al. 2001), a 36 item advanced test of emotional recognition requiring theory of mind and social sensitivity. The Eyes Test measures a participant’s ability to determine complex emotional states from limited information, without context, and is a performance measure of empathy. Participants are required to look at a picture of a person’s eyes and select one of four words that best describe what the person in the picture is feeling. The Eyes Test was developed and validated on a combined group of adults with either HFA or AS compared to typically developed controls. Vellante et al. (2012) meta-analysis demonstrated that the Eyes Test has good internal consistency, *α* = .70 (Dehning et al. 2012), and *α* = .77, using Guttman’s split-half method (Serafin and Surian 2004). Test–retest reliability for the Eyes Test has also been shown to be fair, *ICC* = .65 (Vellante et al. 2012). Finally, The Eyes Test demonstrates diagnostic sensitivity between people who are typically developing and people with either HFA or AS (Baron-Cohen et al. 2001). This is important as it indicates that the test measures some of core features of ASC and therefore is key to understanding differences between HFA and AS.

### Procedure

Once registered with the CARD, participants completed the research centre’s standard registration questions. The questionnaire asked for basic demographic information including age, sex, educational attainments, and employment status. Mandatory fields also include diagnosis, diagnostic method, and comorbid conditions, while general screening questions assessed specific research study inclusion and exclusion criteria, such as medication. Participants then navigate to and selected tasks from the online test battery, completing as many as desired. Each task is preceded by the appropriate instructions and participants are able to log in and out as often as they wish.

As required by the ethical approval for the Cambridge Autism Research Database, local ethical approval was obtained from the University of East Anglia Faculty of Medicine and Health Sciences Research Ethics Committee to gain access parts of the CARD, including the target measures. The dataset was manually searched to ensure that required information (sex, age, diagnosis and Ravens Progressive Matrices data) were available, and that the target measures were complete. Participants with missing data were excluded. This reduced the full dataset of eligible participants from 99 individuals with HFA and 955 individuals with AS to 43 individuals with HFA and 446 individuals with AS. For comparative purposes a random sample, stratified by sex and matching the HFA group (male *n* = 20, female *n* = 23), of 43 participants (male *n* = 20, female *n* = 23) was drawn from the AS group.

### Statistical Analysis

The data, EQ total scores and accuracy scores from the Eyes Test, were analysed using SPSS (SPSS Inc.) software version 18, and inspected for departures from normality. The data were not normally distributed, and therefore, groups were compared using the Mann–Whitney U test. For all comparisons the converted z-score, rather than the U score, is reported so that the results may be readily compared against critical values of a normal distribution. A supplementary analysis using logistic regression, which is not sensitive to departures from normality, was also conducted, using variables that significantly differed between the two groups, in order to explore whether these variables predicted clinical group membership.

## Results

### Descriptive Statistics

The number of men and women within each group was matched. There was no significant difference in age between the HFA group and the AS group, *z* = −.039, *p* = 969 (Table 1). There was no significant difference in IQ, as measured by Raven’s Progressive Matrices (Raven et al. 1997), between the HFA group and the AS group, *z* = −.056, *p* = .955. In fact, although minimal non-significant differences were observed across gender—diagnosis comparisons, on a whole group by group comparison the HFA and AS groups were found to be identical on mean IQ.

### Exploratory Analyses

#### Sex Differences

There was no significant difference in EQ total score between adult men and women with HFA, *z* = −.610, *p* = .542 or between men with AS and women with AS, *z* = −.403, *p* = .687. Similarly, no significant difference was observed on the Eyes Test between men and women with HFA *z* = −.403, *p* = .687 or between men and women with AS, *z* = −.817, *p* = .414.

#### Empathy

No significant difference was found between adults with HFA and adults with AS in how they reported their abilities to empathise with others, as measured by the EQ, *z* = −.926, *p* = .335.

#### Emotion Recognition

**Fig. 1:**
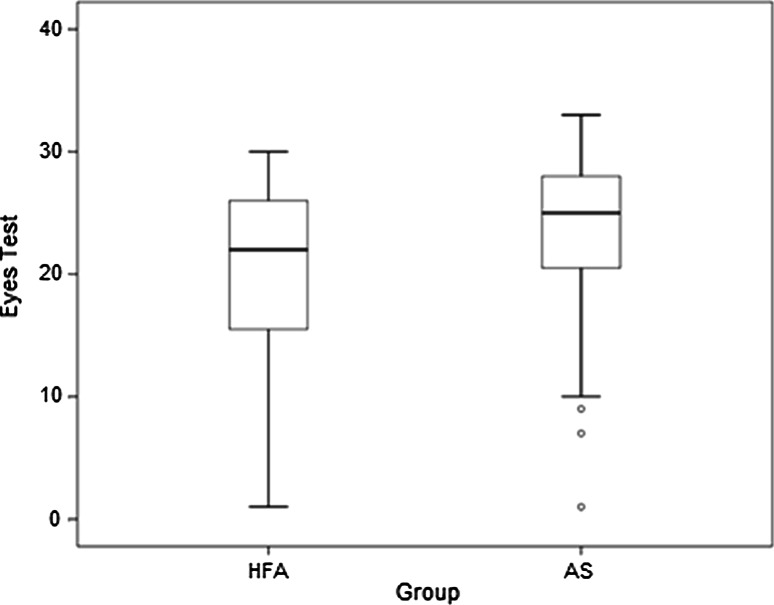
Mean and spread of Eyes Test scores for adults with HFA and AS. *Thick lines* represent the mean scores, the spread of data within the upper and lower quartiles is represented within the *box*, and *vertical lines* represent the full range of scores from the highest to lowest values. Outliers are represented by *circles*

The ability to accurately interpret complex emotional states from expressions in the eyes was explored between groups using the Eyes Test accuracy scores. Adults with AS were significantly better at correctly interpreting complex emotions than adults with HFA, *z* = −2.367, *p* = .018, Cohen’s *d* = .47 (Fig. 1; Table 2).

**Table 2:** Means and medians of main comparisons

	HFA (*n* = 43)	Male (*n* = 20)	Female (*n* = 23)	AS (*n* = 43)	Male (*n* = 20)	Female (*n* = 23)
*M* age (SD)	39.09 (13.05)	44.85 (12.15)	34.09 (11.90)	38.56 (11.92)	41.25 (11.68)	36.22 (11.89)
*M* RPM* (SD)	18.91 (1.74)	19.50 (0.69)	18.39 (2.20)	18.91 (1.73)	18.90 (1.37)	18.91 (2.04)

Measures	*M* (SD)					
EQ	16.91 (10.22)	15.65 (8.91)	18.00 (11.33)	17.98 (8.86)	17.75 (10.14)	18.17 (7.81)
Eyes Test	20.09 (7.66)	19.35 (8.78)	20.74 (6.67)	23.53 (7.00)	24.35 (6.72)	22.83 (7.31)

	Median (range)					
EQ	14.00 (46)	12.00 (37)	17.00 (45)	16.00 (46)	22.50 (36)	21.55 (32)
Eyes Test	22.00 (29)	21.00 (29)	23.00 (23)	25.00 (32)	25.50 (24)	25.00 (31)

RPM = Raven’s Progressive Matrices, EQ = Empathy Quotient, Eyes Test = Reading the Mind in the Eyes Test

### Supplementary Analysis

A significant difference was found in scores on the Eyes Test between adults with HFA and adults with AS. A binary logistic regression was conducted to explore the extent to which scores on the Eyes Test could predict group membership. Performance on the Eyes Test significantly predicted group membership, *χ*^2^(1) = 4.728, *p* = .030, with 59.3 % of cases being accurately predicted, and an observed odds ratio of *Exp*(*B*) = 1.068, 95 % CI [1.004, 1.137].

## Discussion

This study set out to examine whether there were any difference on measures of empathy between two diagnostic categories on the autism spectrum, AS and HFA. No significant difference was observed in self-reported drive to empathise between adults with HFA and adults with AS, matched for age, sex, and IQ. In contrast, adults with AS were significantly better than adults with HFA at correctly interpreting complex emotions in others’ eyes. This observed difference in performance showed a moderate effect size and significantly predicted group membership, albeit with a modest predictive accuracy of 59.3 %, and odds ratio of 1.068, which is small. No significant differences were observed between males and females within the HFA and AS groups on any of the tasks. The overall poor performance observed among participants with both HFA and AS on the EQ and Eyes Test is consistent with previous research (Barnes 2012; Baron-Cohen et al. 2001; Baron-Cohen and Wheelwright 2004; Fabio et al. 2011; Lai et al. 2011, 2012) and supports the mindblindness theory of autism (Baron-Cohen 1995). No significant difference was observed in the reported ability and drive to empathise, measured by the EQ, between adults with HFA or AS and thus difficulties in empathy may be further confirmed as a unifying feature of ASC in adults.

A previous study reports that children with HFA have greater difficulties with theory of mind skills than children with AS (Paynter and Peterson 2010), a difference that was not readily apparent on the EQ in the present study. It is important to note that the lack of difference in self-reported empathy ability does not solely depend on theory of mind skills, as the EQ measures both cognitive and affective empathy; however this difference in child and adult use of theory of mind skills is interesting and there are a number of possible explanations as to why this difference might exist. Some research suggests that the cognitive and behavioural phenotypes associated with ASC are more pronounced in childhood than in adulthood (Howlin et al. 2004). Children with AS, whose language development follows closer to typical trajectories, may have a greater intellectual or linguistic abilities, which enables the faster development of theory of mind skills, than children with HFA who have the additional complications of language delay and associated comprehension difficulties (Howlin 2003). It could therefore be hypothesised that the lack of difference in this skill in adulthood reflects a narrowing of the developmental gap in seen in child populations, a hypothesis that is also supported in neuroanatomical research (Lai et al. 2015). This finding is also linked to the construct of theory of mind, which is a developmental skill and so changes over time (Scheeren et al. 2013). If this is the case, children with HFA specifically may benefit from increased support around developing this skill to better understand social and emotional interactions. Longitudinal studies are needed to test this hypothesis.

In contrast to the EQ result, a significant difference was observed between adults with HFA and adults with AS in their ability to correctly interpret complex emotional states, measured by the Eyes Test. This suggests that non-contextual theory of mind skills are significantly more impaired in adults with HFA compared to adults with AS. The results also indicate that the Eyes Test has some sensitivity to distinguishing between potential clinical subgroups, although it is not a diagnostic test and it is important to note that the predictive accuracy is low. To our knowledge, this is the first evidence for a difference in this profile between adults with HFA and those with AS. Social learning theories highlight the importance of emotion, within social interactions, in facilitating social learning (Treur and van Wissen 2013). Facial expression mimicry during social interactions also enhances social coordination and improves quality of relationships (Hess and Bourgeois 2010). As adults with HFA were less able to correctly interpret complex emotional states in others than adults with AS, it is likely social interactions may be more challenging for people with HFA. This group may therefore need increased social support.

No significant differences were observed between men and women with either HFA or AS, on any of the measures used in this research. This lack of sex difference has recently been replicated in larger samples (Baron-Cohen et al. 2014). Within typically developing populations, women perform significantly better than men on the Eyes Test, a difference which does not exist in ASC populations (Baron-Cohen et al. 1997, 2014). This is also the case on the EQ (Baron-Cohen and Wheelwright 2004; Baron-Cohen et al. 2014). The lack of sex difference on the EQ and the Eyes Test among adults with ASC replicates previous research exploring behavioural differences between men and women with ASC (Lai et al. 2011; Wheelwright et al. 2006). However, as far as we are aware, this is the first time that this sex similarity has been observed on these measures in discrete HFA and AS groups. This finding across groups indicates that the reduced empathising profile is seen in a similar way in adults with HFA and adults with AS. This suggests that the between-groups difference observed on the Eyes Test relates to a fundamental difference between these clinical populations as it is not influenced by sex differences.

The results of this study raise a number of questions about the most clinically helpful way of conceptualising HFA and AS. The significant difference observed between adults with HFA and adults with AS on the Eyes Test is of particular relevance to the debate over whether the conditions should be conceptualised as separate subgroups with overlapping features, as they are in ICD-10 (WHO 1994), or as not having qualitative differences, as they are in DSM-5 (APA 2013). It could be hypothesised that the atypical language acquisition seen in children with HFA, but not in children with AS, leads to difficulties in early social interactions that in turn lead to a weaker ability in identifying complex emotions among adults with HFA, observed in the present study. Longitudinal studies comparing the trajectories of language and social skills development in relation to complex emotion recognition between children with AS and children with HFA would be beneficial.

The Eyes Test has known neuroanatomical correlates including the dorsolateral prefrontal cortex, the left medial frontal cortex, the superior temporal gyrus, and parts of the amygdala (Baron-Cohen et al. 1999; Holt et al. 2014; Richell et al. 2003). If differences exist between adults with HFA and adults with AS on this task, it may reflect underlying neuroanatomical or neuro-functional differences between the conditions. This area of exploration is in its infancy (Lai et al. 2015; McAlonan et al. 2008; Yu et al. 2011) and more studies exploring functional and structural differences between HFA and AS neuroanatomy may help to explain differences in the presentation of the conditions. Based on the results of this study, combined with the neuroanatomical correlates of the Eyes Test, one hypothesis is that HFA and AS differ in the neurological areas that underpin the ability to interpret emotional states. This may mean that differences in abilities between the conditions are more canalised, i.e. they are fundamental characteristics of the populations that are not altered by individual variation, and may explain why the results of the Eyes Test were shown to be predictive of clinical group.

The discrepancy between results on the EQ and the Eyes Test is interesting from a clinical perspective. Self-report of perceived abilities to empathise on the EQ showed no difference between adults with HFA and those with AS. The Eyes Test, an objective measure of the ability to interpret what another person is feeling based on their expression, showed a significant difference between groups, with adults with HFA performing significantly worse than adults with AS. This performance difference suggests that, as well as having greater difficulty interpreting complex emotional states in others, adults with HFA may have less insight into this area of social difficulty than adults with AS do. Adults with HFA are not reporting the additional difficulties on the EQ that we may expect given the observed skill difficulties on the Eyes Test. The implications of a possibly reduced insight into social skill difficulties among adults with HFA need to be considered further in future studies.

The difference between subjective and objective reports of empathy is an area that has been increasingly identified in research across typically developed populations (Devlin et al. 2014; Realo et al. 2003). Devlin et al. (2014) refer to the difference between perceived and actual skills as a ‘belief-ability gap’. In the present study, the observed difference between groups at the behavioural, rather than the self-report level, may also by hypothesised to reflect greater difficulties with mentalizing the self in adults with HFA. Mentalizing refers the ability to think of the experiences of the self and others in interpersonal contexts (Fonagy and Bateman 2006). Lombardo et al. (2007) have observed that individuals with ASC who are more self-focused are better at mentalizing. One hypothesis, therefore, is that individuals with HFA are less self-focused that those with AS and so it is a difficulty in mentalizing that means self-reported empathy is less aligned with actual performance. Further studies in this area are clearly needed.

This study has a number of strengths. First, it contributes to an area which has not received much attention; the direct comparison of social and emotional functioning between adults with HFA and adults with AS. This study also considered both objective and subjective abilities of adults, which has allowed for a practical consideration of the differences between subgroups. Second, the lack of significant difference in IQ between subgroups is important as the reliability of the HFA deficit finding is improved by the removal of general intelligence as a confounding variable. Third, the EQ and Eyes Test have undergone substantial reliability tests and have excellent psychometric properties (Allison et al. 2011; Dehning et al. 2012) although the comparatively weaker, yet still fair, test–retest reliability of the Eyes-Test (ICC = 0.65) should be noted. Overall reliability was, however, enhanced by sufficient sample sizes with sufficient statistical power (Cohen 1988).

Many participants registered with CARD have completed in-person testing for research projects and thus have received confirmatory diagnosis using the Autism Diagnostic Observation Schedule (ADOS; Lord et al. 2000), Autism Diagnostic Interview (ADI; Lord et al. 1994), or DSM-IV diagnostic criteria. Other participants registered through the CARD self-reported a diagnosis. Self-report of official diagnosis within the ASC populations is highly reliable (Auyeung et al. 2012; Daniels et al. 2012), and only participants who had been able to provide information with regards to where and how they received their diagnosis were included in the current study. Nevertheless, the validity of the findings would have been further enhanced if all participants had their diagnostic sub-group independently confirmed with a standardised diagnostic tool.

While the main comparisons provide a useful insight into the similarities and differences between emotional processing across HFA and AS, additional explorations of how factors such as verbal IQ (VIQ) or socio-economic status impact on the findings may have provided interesting data and alternative interpretations of the results. The lack of VIQ data means that the question of whether VIQ can be eliminated as a confounding variable in this study cannot be definitively answered. The results therefore need to be interpreted with some caution, however, the validity of the findings is enhanced by the groups being matched on general intelligence. Furthermore, while previous studies have suggested that the cognitive profiles associated with HFA and AS may be distinguished by VIQ (e.g. Planche and Lemonnier 2012), there are equal studies that show no VIQ differences between groups (e.g. Spek et al. 2008). The inconsistency in these data suggests individual variation in cognitive profiles, not easily unified into associated diagnostic categories, which reduces the potential of a between groups VIQ confound. Additionally, both the EQ and the Eyes Test require a significant amount of language comprehension for completion and both were validated on a mixed group of adults with AS and adults with HFA, without impact on the psychometric properties of the measures being identified. Finally, given the language demands of the EQ and the Eyes Test, if VIQ differences between groups existed, difficulties in completion across both tasks would be expected in the HFA group, and this was not observed. Further studies that include a comparison of VIQ impact may, however, help to enhance our understanding of these observed differences between HFA and AS.

This study found that adults with HFA are significantly more impaired than adults with AS at correctly identifying complex emotional states in others, using the Eyes Test. To our knowledge, this is the first time this difference has been observed. While it is not possible to draw broad conclusions from a single study, the findings suggest a difference between groups and a need for the subtleties in presentation between HFA and AS to be considered further, so that any impact on everyday life can be understood and support can be tailored appropriately. Future studies exploring the impact of language on social-emotional functioning between these groups are indicated and differences between cognitive and affective empathy should be explored further. While the diagnostic conceptualisation of ASC remains complex, the differences observed here highlight a possible beneficial role of some subgrouping within ASC.

## References

[CR2] Allison C, Baron-Cohen S, Wheelwright SJ, Stone MH, Muncer SJ (2011). Psychometric analysis of the Empathy Quotient (EQ). Personality and Individual Differences.

[CR3] Amaral DG, Schumann CM, Wu Nordahl C (2008). Neuroanatomy of autism. Trends in Neurosciences.

[CR4] American Psychiatric Association (APA). (2000). *Diagnostic and statistical manual of mental disorders* (4th ed., Text Revision). Washington, DC: APA.

[CR5] American Psychiatric Association (APA). (2013). *Diagnostic and statistical manual of mental disorders* (5th ed., Text Revision). Arlington, VA: APA.

[CR6] Auyeung B, Allison C, Wheelwright S, Baron-Cohen S (2012). Brief report: Development of the adolescent Empathy and Systemizing Quotients. Journal of Autism and Developmental Disorders.

[CR7] Barnes JL (2012). Fiction, imagination, and social cognition: Insights from autism. Poetics.

[CR8] Baron-Cohen S (1995). Mindblindness: An essay on autism and theory of mind.

[CR9] Baron-Cohen S (2002). The extreme male brain theory of autism. Trends in Cognitive Sciences.

[CR10] Baron-Cohen S (2011). Zero degrees of empathy: A new theory of human cruelty.

[CR11] Baron-Cohen, S., Bowen, D. C., Holt, R. J., Allison, C., Auyeung, B., Lombardo, M. V., et al. (2015). The “Reading the Mind in the Eyes” test: Complete absence of typical sex difference in ~400 men and women with autism. *PLoS ONE,**10*(8). doi:10.1371/journal.pone.0136521. 10.1371/journal.pone.0136521 PMC4552377 26313946

[CR12] Baron-Cohen, S., Cassidy, S., Auyeung, B., Allison, C., Achoukhi, M., Robertson, S., et al. (2014). Attenuation of typical sex differences in 800 adults with autism vs. 3,900 controls. *PLoS ONE,**9*(7). 10.1371/journal.pone.0102251 PMC4100876 25029203

[CR13] Baron-Cohen S, Jolliffe T, Mortimore C, Robertson M (1997). Another advanced test of theory of mind: Evidence from very high functioning adults with autism or Asperger syndrome. Journal of Child Psychology and Psychiatry.

[CR14] Baron-Cohen S, Leslie AM, Frith U (1985). Does the autistic child have a “theory of mind”?. Cognition.

[CR15] Baron-Cohen S, Ring HA, Wheelwright S, Bullmore ET, Brammer MJ, Simmons A, Williams SC (1999). Social intelligence in the normal and autistic brain: An fMRI study. European Journal of Neuroscience.

[CR16] Baron-Cohen S, Wheelwright S (2004). The Empathy Quotient: An investigation of adults with Asperger syndrome or high functioning autism, and normal sex differences. Journal of Autism and Developmental Disorders.

[CR17] Baron-Cohen S, Wheelwright S, Hill J, Raste Y, Plumb I (2001). The “Reading the Mind in the Eyes” Test revised version: A study with normal adults, and adults with Asperger syndrome or high-functioning autism. Journal of Child Psychology and Psychiatry and Allied Disciplines.

[CR18] Bedford R, Elsabbagh M, Gliga T, Pickles A, Senju A, Charman T, Johnson MH (2012). Precursors to social and communication difficulties in infants at-risk for autism: Gaze following and attentional engagement. Journal of Autism and Developmental Disorders.

[CR19] Cassidy S, Bradley P, Robinson J, Allison C, McHugh M, Baron-Cohen S (2014). Suicidal ideation and suicide plans or attempts in adults with Asperger’s syndrome attending a specialist diagnostic clinic: A clinical cohort study. The Lancet Psychiatry.

[CR20] Cohen J (1988). Statistical power analysis for the behavioral sciences.

[CR21] Daniels AM, Rosenberg RE, Anderson C, Law JK, Marvin AR, Law PA (2012). Verification of parent-report of child autism spectrum disorder diagnosis to a web-based autism registry. Journal of Autism and Developmental Disorders.

[CR22] Dehning S, Girma E, Gasperi S, Meyer S, Tesfaye M, Siebeck M (2012). Comparative cross-sectional study of empathy among first year and final year medical students in Jimma University, Ethiopia: Steady state of the heart and opening of the eyes. BMC Medical Education.

[CR23] Devlin, H. C., Zaki, J., Ong, D. C., & Gruber, J. (2014). Not as good as you think? Trait positive emotion is associated with increased self-reported empathy but decreased empathic performance. *PLoS ONE, 9*(10). 10.1371/journal.pone.0110470 PMC4212943 25353635

[CR24] Fabio RA, Oliva P, Murdaca AM (2011). Systematic and emotional contents in overselectivity processes in autism. Research in Autism Spectrum Disorders.

[CR26] Fonagy P, Bateman AW (2006). Mechanisms of change in mentalization-based treatment of BPD. Journal of Clinical Psychology.

[CR27] Ghaziuddin M (2010). Brief Report: Should DSM V drop Asperger syndrome?. Journal of Autism and Developmental Disorders.

[CR100] Ghaziuddin M, Mountain-Kimchi K (2004). Defining the intellectual profile of Asperger syndrome: Comparison with high-functioning autism. Journal of Autism and Developmental Disorders.

[CR28] Goldstein TR, Winner E (2012). Enhancing empathy and theory of mind. Journal of Cognition and Development.

[CR29] Gotham K, Bishop SL, Brunwasser S, Lord C (2014). Rumination and perceived impairment associated with depressive symptoms in a verbal adolescent–adult ASD sample. Autism Research.

[CR30] Groen WB, Zwiers MP, van der Gaag RJ, Buitelaar JK (2008). The phenotype and neural correlates of language in autism: An integrative review. Neuroscience and Biobehavioral Reviews.

[CR31] Hess U, Bourgeois P (2010). You smile-I smile: Emotion expression in social interaction. Biological Psychology.

[CR32] Holt RJ, Chura LR, Lai MC, Suckling J, von dem Hagen E, Calder AJ (2014). ‘Reading the Mind in the Eyes’: An fMRI study of adolescents with autism and their siblings. Psychological Medicine.

[CR33] Howlin P (2003). Outcome in high-functioning adults with autism with and without early language delays: Implications for the differentiation between autism and Asperger syndrome. Journal of Autism and Developmental Disorders.

[CR34] Howlin P, Goode S, Hutton J, Rutter M (2004). Adult outcome for children with autism. Journal of Child Psychology and Psychiatry and Allied Disciplines.

[CR35] Kaland N (2011). Brief report: Should Asperger syndrome be excluded from the forthcoming DSM-V?. Research in Autism Spectrum Disorders.

[CR36] Lai M-C, Lombardo MV, Chakrabarti B, Baron-Cohen S (2013). Subgrouping the autism “spectrum”: Reflections on DSM-5. PLoS Biology.

[CR37] Lai, M. C., Lombardo, M. V., Ecker, C., Chakrabarti, B., Suckling, J., Bullmore, E. T., et al. (2015). Neuroanatomy of individual differences in language in adult males with autism. *Cerebral Cortex,**25*(10), 3613–3628. doi:10.1093/cercor/bhu211. 10.1093/cercor/bhu211 PMC4585508 25249409

[CR38] Lai, M.-C., Lombardo, M. V., Pasco, G., Ruigrok, A. N. V., Wheelwright, S. J., Sadek, S. A, Chakrabarti, B., et al. (2011). A behavioral comparison of male and female adults with high functioning autism spectrum conditions. *PloS One, 6*(6). 10.1371/journal.pone.0020835 PMC3113855 21695147

[CR39] Lai, M. C., Lombardo, M. V., Ruigrok, A. N., Chakrabarti, B., Wheelwright, S. J., Auyeung, B., et al. (2012). Cognition in males and females with autism: similarities and differences. *PLoS One*, *7*(10). 10.1371/journal.pone.0047198 PMC3474800 23094036

[CR40] Lawrence EJ, Shaw P, Baker D, Baron-Cohen S, David AS (2004). Measuring empathy: Reliability and validity of the Empathy Quotient. Psychological Medicine.

[CR41] Lombardo MV, Barnes JL, Wheelwright SJ, Baron-Cohen S (2007). Self-referential cognition and empathy in autism. PLoS ONE.

[CR101] Lord C, Rutter M, Le Couteur A (1994). Autism Diagnostic Interview–Revised: A revised version of a diagnostic interview for caregivers of individuals with possible pervasive developmental disorders. Journal of Autism and Developmental Disorders.

[CR102] Lord C, Risi S, Lambrecht L, Cook EH, Leventhal BL, DiLavore PC (2000). The autism diagnostic observation schedule-generic: A standard measure of social and communication deficits associated with the spectrum of autism. Journal of Autism and Developmental Disorders.

[CR42] Mansell J, Elliott T, Beadle-Brown J, Ashman B, Macdonald S (2002). Engagement in meaningful activity and “active support” of people with intellectual disabilities in residential care. Research in Developmental Disabilities.

[CR43] Matson JL, Boisjoli JA (2008). Strategies for assessing Asperger’s syndrome: A critical review of data based methods. Research in Autism Spectrum Disorders.

[CR44] Mazza M, Pino MC, Mariano M, Tempesta D, Ferrara M, De Berardis D (2014). Affective and cognitive empathy in adolescents with autism spectrum disorder. Frontiers in Human Neuroscience.

[CR45] McAlonan GM, Suckling J, Wong N, Cheung V, Lienenkaemper N, Cheung C, Chua SE (2008). Distinct patterns of grey matter abnormality in high-functioning autism and Asperger’s syndrome. Journal of Child Psychology and Psychiatry.

[CR46] McConnell SR (2002). Interventions to facilitate social interaction for young children with Autism: Review of available research and recommendations for educational intervention and future research. Journal of Autism and Developmental Disorders.

[CR47] Ozonoff S, Rogers SJ, Pennington BF (1991). Asperger’s syndrome: Evidence of an empirical distinction from high-functioning autism. Journal of Child Psychology and Psychiatry.

[CR48] Ozonoff S, South M, Miller JN (2000). DSM-IV-defined Asperger syndrome: Cognitive, behavioral and early history differentiation from high-functioning autism. Autism.

[CR49] Palmen A, Didden R, Lang R (2012). A systematic review of behavioral intervention research on adaptive skill building in high-functioning young adults with autism spectrum disorder. Research in Autism Spectrum Disorders.

[CR50] Paynter J, Peterson C (2010). Language and ToM development in autism versus Asperger syndrome: Contrasting influences of syntactic versus lexical/semantic maturity. Research in Autism Spectrum Disorders.

[CR51] Pina-Camacho L, Villero S, Boada L, Fraguas D, Janssen J, Mayoral M (2013). Structural magnetic resonance imaging data do not help support DSM-5 autism spectrum disorder category. Research in Autism Spectrum Disorders.

[CR52] Planche P, Lemonnier E (2012). Research in autism spectrum disorders Children with high-functioning autism and Asperger’s syndrome: Can we differentiate their cognitive profiles ?. Research in Autism Spectrum Disorders.

[CR53] Premack D, Woodruff G (1978). Does the chimpanzee have a ‘theory of mind’?. Behavioral and Brain Sciences.

[CR54] Raven JC, Raven J, Court JH (1997). Manual for Raven’s progressive matrices and vocabulary scales.

[CR55] Realo A, Allik J, Nõlvak A, Valk R, Ruus T, Schmidt M, Eilola T (2003). Mind-reading ability: Beliefs and performance. Journal of Research in Personality.

[CR56] Richell RA, Mitchell DGV, Newman C, Leonard A, Baron-Cohen S, Blair RJR (2003). Theory of mind and psychopathy: Can psychopathic individuals read the “language of the eyes”?. Neuropsychologia.

[CR57] Rogers SJ (2009). What are infant siblings teaching us about autism in infancy?. Autism Research.

[CR58] Schalock RL (2004). The concept of quality of life: What we know and do not know. Journal of Intellectual Disability Research.

[CR59] Scheeren AM, de Rosnay M, Koot HM, Begeer S (2013). Rethinking theory of mind in high-functioning autism spectrum disorder. Journal of Child Psychology and Psychiatry.

[CR60] Schultz RT (2005). Developmental deficits in social perception in autism: The role of the amygdala and fusiform face area. International Journal of Developmental Neuroscience.

[CR61] Serafin M, Surian F (2004). Il Test degli Occhi: uno strumento per valutare la ‘‘teoria della mente’’. [Italian]. Giornale Italiano di Psicologia.

[CR62] Spek A, Scholte EM, van Berckelaer-Onnes I (2008). Brief report: The use of WAIS-III in adults with HFA and Asperger syndrome. Journal of Autism and Developmental Disorders.

[CR63] Sucksmith E, Allison C, Baron-Cohen S, Chakrabarti B, Hoekstra RA (2013). Empathy and emotion recognition in people with autism, first-degree relatives, and controls. Neuropsychologia.

[CR64] Tobin MC, Drager KDR, Richardson LF (2014). A systematic review of social participation for adults with autism spectrum disorders: Support, social functioning, and quality of life. Research in Autism Spectrum Disorders.

[CR65] Treur J, van Wissen A (2013). Conceptual and computational analysis of the role of emotions and social influence in learning. Procedia—Social and Behavioral Sciences.

[CR66] Tsai LY, Ghaziuddin M (2014). DSM-5 moves forward into the past. Journal of Autism and Developmental Disorders.

[CR67] Vellante M, Baron-Cohen S, Melis M, Marrone M, Petretto DR, Masala C, Preti A (2012). The “Reading the Mind in the Eyes” test: systematic review of psychometric properties and a validation study in Italy. Cognitive Neuropsychiatry.

[CR68] Volkmar FR, Partland JC (2014). From Kanner to DSM-5: Autism as an evolving diagnostic concept. Annual Review of Clinical Psychology.

[CR69] Wheelwright S, Baron-Cohen S, Goldenfeld N, Delaney J, Fine D, Smith R (2006). Predicting Autism Spectrum Quotient (AQ) from the Systemizing Quotient-Revised (SQ-R) and Empathy Quotient (EQ). Brain Research.

[CR70] Williams DL, Goldstein G, Kojkowski N, Minshew NJ (2008). Do individuals with high functioning autism have the IQ profile associated with nonverbal learning disability?. Research in Autism Spectrum Disorders.

[CR71] Wilson CE, Happé F, Wheelwright SJ, Ecker C, Lombardo MV, Johnston P (2014). The neuropsychology of male adults with high-functioning autism or Asperger syndrome. Autism Research.

[CR72] Wing L, Gould J (1979). Severe impairments of social interaction and associated abnormalities in children: Epidemiology and classification. Journal of Autism and Developmental Disorders.

[CR74] Witwer AN, Lecavalier L (2008). Examining the validity of autism spectrum disorder subtypes. Journal of Autism and Developmental Disorders.

[CR75] World Health Organization (WHO) (1994). The ICD-10 classification of mental and behavioural disorders. Clinical descriptions and guidelines.

[CR76] Yu KK, Cheung C, Chua SE, McAlonan GM (2011). Can Asperger syndrome be distinguished from autism? An anatomic likelihood meta-analysis of MRI studies. Journal of Psychiatry and Neuroscience.

